# Factors related to preparedness for emergency hemodialysis in the event of a natural disaster: Erratum

**DOI:** 10.1097/MD.0000000000030550

**Published:** 2022-09-02

In the article, “Factors related to preparedness for emergency hemodialysis in the event of a natural disaster”,^[1]^ which appears in Volume 101, Issue 24 of *Medicine*, information appeared incorrectly in Table 4. Originally, the information in row Availability of work and column Comparison category/base category appeared as 0: Working, 1: Not working. This has been corrected to 0: Not Working, 1: Working.
